# Supplementation of *Syzygium cumini* seed powder prevented obesity, glucose intolerance, hyperlipidemia and oxidative stress in high carbohydrate high fat diet induced obese rats

**DOI:** 10.1186/s12906-017-1799-8

**Published:** 2017-06-02

**Authors:** Anayt Ulla, Md Ashraful Alam, Biswajit Sikder, Farzana Akter Sumi, Md Mizanur Rahman, Zaki Farhad Habib, Mostafe Khalid Mohammed, Nusrat Subhan, Hemayet Hossain, Hasan Mahmud Reza

**Affiliations:** 1 Department of Pharmaceutical Sciences, North South University, Dhaka, -1229 Bangladesh; 2 BCSIR Laboratories, Bangladesh Council of Scientific and Industrial Research (BCSIR), Dhaka, Bangladesh

**Keywords:** High carbohydrate high fat diet, Dyslipidemia, Fibrosis, Oxidative stress, Antioxidant

## Abstract

**Background:**

Obesity and related complications have now became epidemic both in developed and developing countries. Cafeteria type diet mainly composed of high fat high carbohydrate components which plays a significant role in the development of obesity and metabolic syndrome.

This study investigated the effect of *Syzygium cumini* seed powder on fat accumulation and dyslipidemia in high carbohydrate high fat diet (HCHF) induced obese rats.

**Method:**

Male Wistar rats were fed with HCHF diet ad libitum, and the rats on HCHF diet were supplemented with *Syzygium cumini* seed powder for 56 days (2.5% *w*/w of diet). Oral glucose tolerance test, lipid parameters, liver marker enzymes (AST, ALT and ALP) and lipid peroxidation products were analyzed at the end of 56 days. Moreover, antioxidant enzyme activities were also measured in all groups of rats.

**Results:**

Supplementation with *Syzygium cumini* seed powder significantly reduced body weight gain, white adipose tissue (WAT) weights, blood glucose, serum insulin, and plasma lipids such as total cholesterol, triglyceride, LDL and HDL concentration. *Syzygium cumini* seed powder supplementation in HCHF rats improved serum aspartate amino transferase (AST), alanine amino transferase (ALT), and alkaline phosphatase (ALP) activities. *Syzygium cumini* seed powder supplementation also reduced the hepatic thiobarbituric acid reactive substances (TBARS) and elevated the antioxidant enzyme superoxide dismutase (SOD) and catalase (CAT) activities as well as increased glutathione (GSH) concentration. In addition, histological assessment showed that *Syzygium cumini* seed powder supplementation prevented inflammatory cell infiltration; fatty droplet deposition and fibrosis in liver of HCHFD fed rats.

**Conclusion:**

Our investigation suggests that *Syzygium cumini* seed powder supplementation prevents oxidative stress and showed anti-inflammatory and antifibrotic activity in liver of HCHF diet fed rats. In addition, *Syzygium cumini* seed powder may be beneficial in ameliorating insulin resistance and dyslipidemia probably by increasing lipid metabolism in liver of HCHF diet fed rats.

## Background

Obesity is one of the most prevalent heath conditions which may foster various diseases such as dyslipidemia, insulin resistance, hypertension and increased risks of cardiovascular mortality [1]. Being overweight is not the only problem in obesity but the accumulation of excess dietary calories into visceral fat and the release of high concentrations of free fatty acids into various organs eventually lead towards metabolic syndrome. According to the world health organization it has been defined as a “medical condition in which excess body fat is accumulated to the extent that can effect negatively on health”. It is a chronic, multifactorial and complex disease resulting from a long-term imbalance between the energy intake and expenditure, however, genetic, physiological, behavioral and environmental factors are also involved [2]. There is presently a global epidemic of obesity found in all age groups and in both developed and developing countries. The worldwide prevalence of obesity is more than doubled between 1980 and 2014. Currently, more people die due to being overweight than being underweight in the world (WHO 2015). Hence, obesity is an increasing concern of society which reduces overall quality of life and leads to premature death. A large body of evidences also indicate that the global epidemic of obesity is fuelled by the societal factors that promote sedentary lifestyle and the consumption of high-fat, energy-dense diets for global epidemic of obesity [3].

Furthermore, it is also found that obesity predisposes the condition of oxidative stress which further leads to the various complications like endothelial dysfunction, nonalcoholic fatty liver disease, microvascular complications and nephropathy [4, 5]. Multiple mechanisms may be involved for oxidative stress condition due to obesity. Some of them are oxidation of fatty acid by mitochondria and peroxisome, lipid rich content diet and overconsumption of oxygen etc. Generally the obese people possesses low antioxidant defense than the normal weight people due to diminished production of antioxidant enzymes like superoxide dismutase (SOD), catalase (CAT) and glutathione peroxidase (GPx). With the increase of central obesity, the antioxidant defense further decreases in an inversely proportional fashion. Obesity is also characterized by enhanced levels of reactive oxygen species (ROS) or reactive nitrogen species (RNS). Studies showed that the hepatic inflammation caused by obesity may also promote tumor formation in dietary induced obese mice [6].

*Syzygium cumini* is an evergreen tree habitat in Bangladesh, India, Eastern Africa, South America and Madagascar. Traditionally the *Syzygium cumini* fruits, leaves, seeds, and bark are used in ayurvedic medicine [7]. *Syzygium cumini* is used for the cure of various diseases including cough, diabetes, dysentery, inflammation and ringworm [7]. Bark decoctions are taken for asthma and bronchitis, used as mouth wash for the astringent effect on mouth ulcerations, and spongy gums and for stomatitis [8]. Gallic acid, ellagic acid, ellagitannins, isoquercetin, quercetin, caffeic acid, ferulic acid, guaiacol, resorcinaldimethyl ether, lignaglucoside, veratrole, β–sitosterol and palmitic acid are isolated from the seed of *Eugenia jambolana* [7]. *Syzygium cumini extract* showed anti-inflammatory activity in animal model [9, 10]. *Syzygium cumini* extract also reduced the production of prostaglandin E_2_, serotonin, and histamine in vivo [11]. Previous report also suggests that *Syzygium cumini* fruit extract possesses hypoglycemic action in diabetic rats [12]. The seed powder of *Syzygium cumini* is also reported to have hypoglycemic action in streptozotocin induced diabetic rats [13]. *Syzygium cumini* extract also protected the oxidative stress by improving antioxidant enzymes in diabetic rats [14]. Earlier report also suggests that *Syzygium cumini* extract may prevent hepatic damage in carbon tetrachloride induced rats [15]. Flavonoid-rich extract of *Eugenia jambolana* seed reduced total cholesterol, LDL-cholesterol, and triacylglycerol while raised HDL-cholesterol levels in diabetic mice [13]. A recent investigation reported that *Syzygium cumini* leaves extract improves peripheral insulin sensitivity, stimulates β-cell insulin release, lowered body weight gain, body mass index, and white adipose tissue mass in monosodium glutamate induced obese rats [16]. Gallic acid and ellagic acid were found to improve metabolic dysfunction and dyslipidemia in high fat diet fed rats [17, 18]. However, no study has reported the anti-obesity, anti-inflammatory and anti-fibrotic role of *Syzygium cumini* seeds extract in high fat diet induced obese animal. Thus the current investigation was undertaken to evaluate the effect of *Syzygium cumini* seed powder supplementation in hepatic fibrosis and inflammation in diet induced obese rats.

## Materials

### Chemicals

The beef tallow was used as a source of high fat in the diet which was obtained from the local beef market and processed well by heating to solidify it for using in the high carbohydrate high fat (HCHF) diet formulation. Thiobarbituric acid (TBA) was purchased from Sigma Chemical Company (USA). Reduced glutathione (GSH) was purchased from J.I. Baker (USA). Alanine aminotransferase (ALT), aspartate aminotransferase (AST), alkaline phosphatase (ALP), triglyceride liquid, Cholesterol (total) liquid, LDL and HDL assay kits were obtained from DCI diagnostics (Budapest, Hungary). All other chemicals and reagents used were of analytical grade.

### Plant material

*Syzygium cumini* seeds were collected from the local market of Dhaka, Bangladesh. *Syzygium cumini* seed was authenticated by Dr. Bokhtiar Uddin, Associate Professor and botanist, Chittagong University, Chittagong, Bangladesh and a catalog accession number was assigned (SBU 119). Then the seeds were undergone processes including removal of the extra pulp and drying. The dry seeds were then grinded to fine powder and mixed well with powdered chow food for using as a supplement.

*Syzygium cumini* seeds powder (20 g) was also used to prepare crude extract using ethanol as solvents in a Soxhlet extractor (temperature 45 °C). This ethanol extract was then used for phenolic content analysis.

### HPLC detection and quantification of polyphenolic compounds

Detection and quantification of selected phenolic compounds in the ethanol extract were determined by HPLC-DAD analysis followed by as previously described method [19]. It was carried out on a Dionex UltiMate 3000 system equipped with quaternary rapid separation pump (LPG-3400RS) and photodiode array detector (DAD-3000RS). Separation was performed using Acclaim® C_18_ (5 μm) Dionex column (4.6 × 250 mm) at 30 °C with a flow rate of 1 ml/min and an injection volume of 20 μl.

### Composition of foods used in this study

Two types of foods were used in this study. One of them was the normal laboratory chow food composed of mainly wheat, wheat bran, rice polishing and fish meal (Table 1). Normal chow diet contained calories as percentage, e. g. 14% proteins, 57% carbohydrates, 13.5% fat. The other type of diet was high carbohydrate high fat diet (HCHF), mainly composed of chow food, sugar, beef tallow and condensed milk. HCHF diet contained calories as percentage, e. g. 14% proteins, 37% carbohydrates, 48% fat.

**Table 1:** Composition of normal and high carbohydrate high fat diet used in this study (for 100 g)

Ingredients of normal lab diet	Percent	Ingredients of HCHF diet	Percent
Wheat	40%	Powdered normal rat feed	15.5%
Wheat bran	20%	Sugar	17.5%
Rice Polishing	5.5%	Beef tallow (fat)	20.0%
Fish meal	10.0%	Condensed milk	39.5%
Oil cake	6.0%	Vit-B complex	0.1%
Gram	0.39%	Salt	0.5%
Pulses	0.39%	Water	100 ml
Milk	0.38%		
Soybean Oil	1.5%		
Molasses	0.095%		
Salt	0.095%		
Embavit (vitamin)	0.1%		

### Animals and treatment

All experimental protocols were approved by the Ethical Committee of North South University for animal care and experimentation. Twenty eight Wistar male rats (Ten to twelve weeks old, 185–200 g) were obtained from Animal production unit of Animal House at Department of Pharmaceutical Sciences, North South University and kept in individual cages at temperature controlled room with a 12 h dark/light cycles environment having free access to standard laboratory feed and water. To study the effects of high carbohydrate high fat diet and its attenuation by supplementation of jam seed, rats were randomly divided into four experimental groups (*n* = 7 each), control (group I), control + *Syzygium cumini* seed (group II), HCHF (Group III) and HCHF+ *Syzygium cumini* (Group IV). Animals of group-I were given the normal laboratory food and water every day for the whole of the study period (8 weeks). Group II received similar treatment as of group I; however, this group was supplemented with *Syzygium cumini* seed powder every day for 8 weeks. Animals of group III received only HCHF treatment, however animals of group IV received both HCHF treatment for 8 weeks and *Syzygium cumini* seed powder mixed in food supplementation every days (2.5% of food, *w*/w). HCHF diet was prepared in our laboratory in pellet forms (Table 1). To assess the glycemic activity before and after the HCHF feeding, OGTT was performed for all four groups before and after finishing of treatment of HCHF. Measurements of body weight and food and water intakes were taken daily.

### Oral glucose tolerance test

At the end of the feeding protocol, rats were kept starved overnight (12 h) and an oral glucose tolerance test was performed. Normal water was supplied during the food deprivation period. Basal blood glucose concentrations were measured in blood taken from the tail vein using (Bionim Corporation, Bedford, MA, USA). The rats were administered 2 g/kg body weight of glucose as a 40% aqueous solution via oral gavage. Tail vein blood samples were taken at 30, 60, 90 and 120 min following glucose administration.

### Animal sacrifice and sample collection

At the end of 8 weeks, all animals were weighed and sacrificed under high dose pentobarbitone sodium (90 mg/kg) anesthesia. Immediately after the sacrifice, blood sample was drawn from abdominal aorta from each rats and placed in citrate buffer containing tubes. Collected blood samples were centrifuged at 8000 rpm and separated the plasma and stored in refrigerator at −20 °C for further analysis. All internal organs such as heart, kidney, spleen and liver were also harvested. Immediately after collection of the organs, they were weighed and stored in neutral buffered formalin (pH 7.4) for histological analysis and in refrigerator at −20 °C for further analysis.

### Plasma biochemistry

Blood was centrifuged at 8000 rpm for 15 min within 30 min of collection into citrate buffer containing tubes. Plasma was separated and transferred to Eppendorff tubes for storage at −20 °C before analysis. Plasma concentrations of total cholesterol, triglycerides, LDL, HDL and activities of plasma alanine transaminase (ALT), aspartate transaminase (AST) and alkaline phosphatase (ALP) were determined using kits supplied by Diatec diagnostic kits (Hungary) according to manufacturer-provided standards and protocols. Plasma insulin was also estimated using insulin kit obtained from Diatec diagnostic kits (Hungary) according to the manufacturer’s protocol.

### Preparation of tissue sample for the assessment of oxidative stress markers

For determination of oxidative stress markers, liver tissue was homogenized in 10 volumes of Phosphate buffer containing (pH 7.4) and centrifuged at 8000 rpm for 15/30 min at 4 °C. The supernatant was collected and used for the determination of protein and enzymatic studies as described below.

### Estimation of lipid peroxidation

Lipid peroxidation in liver was estimated calorimetrically measuring thiobarbituric acid reactive substances (TBARS) followed by previously described method [20]. Lipid peroxidation in the sample was estimated by using 0.1 ml of tissue homogenate (Tris-Hcl buffer, pH 7.5), which was further treated with 2 ml of (1:1:1 ratio) TBA-TCA-HCl reagent (thiobarbituric acid 0.37%, 0.25 N HCl and 15% TCA). This solution was then taken in sealed eppendorf tube and placed in hot water bath for 15 min and cooled in room temperature. The absorbance of clear supernatant was measured against reference blank at 535 nm. MDA concentration was measured using a MDA standard curve straight-line equation. MDA concentration was expressed as nmol/mL or nmol/g tissues.

### Assay of nitric oxide (NO)

Nitric oxide (NO) was determined according to the method described by Tracey et al. as nitrate [21] using Griess reagents. In this study, Griess-Illosvoy reagent was modified by using naphthyl ethylene diamine dihydrochloride (0.1% *w*/*v*) instead of 1-napthylamine (5%). Tissue homogenates (2 mL) and phosphate buffer saline (0.5 mL) were incubated with the reaction mixture (3 mL) at 25 °C for 150 min. A pink colored chromophore was formed. The absorbance of these solutions was measured at 540 nm against the corresponding blank solutions. NO level was measure by using standard curve and expressed as nmol/ml or nmol/g of tissue.

### Advanced oxidation protein products (APOP) assay

Determination of APOP level was performed by modification of the method of Witko-Sarsat et al. [22] and Tiwari et al. [23]. Two mL of plasma was diluted 1: 5 in PBS. Potassium iodide (0.1 mL of 1.16 M) was then added to each tube, followed by 0.2 mL acetic acid after 2 min. The absorbance of the reaction mixture was immediately read at 340 nm against a blank containing 2 mL of PBS, 0.1 mL of KI, and 0.2 mL of acetic acid. The chloramine-T absorbance at 340 nm was found linear within the range of 0 to 100 nmol/mL, AOPP concentrations were expressed as nmol·mL − 1 chloramine-T equivalents.

### Catalase assay (CAT)

CAT activities were determined using previously described method by Chance and Maehly [24]. The reaction solution of CAT activities contained: 2.5 ml of 50 mmol phosphate buffer (pH 5.0), 0.4 ml of 5.9 mmol H_2_O_2_ and 0.1 ml tissue homogenates. Changes in absorbance of the reaction solution at 240 nm were determined after one minute. One unit of CAT activity was defined as an absorbance change of 0.01 as units/min.

### Estimation of superoxide dismutase (SOD) activity

SOD was assayed in plasma and tissue homogenates by using previously described method [25]. Three ml reaction mixture consisted of aliquot of tissue homogenates and PBS to make up the volume to 2.94 ml. The reaction was started by addition of 0.06 ml of 15 mM epinephrine. Change in absorbance was recorded at 480 nm for one min at 15 s interval. Control consisting of all the ingredients, except tissue homogenates, was run simultaneously. One unit of enzyme activity has been defined to cause 50% inhibition of auto-oxidation of epinephrine present in the assay system.

### Reduced glutathione assay (GSH)

Reduced glutathione was estimated by the method of Jollow et al. [26]. Tissue homogenate (1.0 ml) was precipitated with 1.0 ml of (4%) sulfosalicylic acid. The samples were kept at 4 °C for 1 h and then centrifuged at 1200×g for 20 min at 4 °C. The total volume of 3.0 ml assay mixture was composed of 0.1 ml tissue homogenate, 2.7 ml phosphate buffer (0.1 M, pH 7.4) and 0.2 ml DTNB (5,5-dithiobis-2-nitrobenzoic acid), (100 mM). The yellow color of the mixture was developed which was read immediately at 412 nm on a Smart SpecTM plus Spectrophotometer and expressed as ng/mg protein.

### Estimation of myloperoxidase (MPO) activity

MPO activity was determined by a dianisidine-H_2_O_2_ method [27], modified for 96-well plates. Briefly, plasma sample (10 μg protein) was added in triplicate to 0.53 mM *o*-dianisidine dihydrochloride (Sigma) and 0.15 mM H_2_O_2_ in 50 mM potassium phosphate buffer (pH 6.0). The change in absorbance was measured at 460 nm. Results were expressed as units of MPO/mg protein.

### Histopathalogical determination

For microscopic evaluation liver tissues were fixed in neutral buffered formalin and embedded in paraffin, sectioned at 5 μm and subsequently stained with hematoxylin/eosin to see the architecture of hepatic tissue and inflammatory cell infiltration. Sirius red staining for fibrosis and Prussian blue staining for iron deposition were also done in liver sections. Milligan trichrome staining was done for estimation of collagen deposition. Sections were then studied and photographed under light microscope (Zeiss Axioscope) at 40X magnifications**.**

### Statistical analysis

All values are expressed as mean ± standard error of mean (SEM). The results were evaluated by the One-way ANOVA followed by Newman- Keuls post hoc test using Graph Pad Prism Software (USA). Statistical significance was considered at *p* < 0.05 in all cases.

## Results

### Analysis of ethanol extract of *Syzygium cumini seed* powder by HPLC-DAD system

**Fig. 1:**
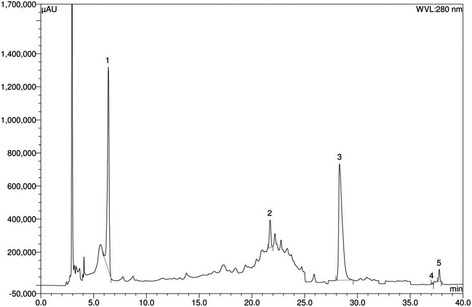
HPLC chromatogram of ethanol extract of *Syzygium cumini*. Peaks: 1, Gallic acid; 2, (−)-epicatechin; 3, ellagic acid; 4, quercetin; 5, *trans*-cinnamic acid

Ethanol extract of *Syzygium cumini seed* powder possesses high amount of gallic acid (1698.37 mg/100 g of dry extract) and ellagic acid (1275.36 mg/100 g of extract) (Fig. 1; Table 2). Notable amount of (−)-epicatechin (258.22 mg/100 g of dry extract) is also present in the extracts (Table 1).

**Table 2:** Contents of polyphenolic compounds in the ethanol extract of *Syzygium cumini* (*n* = 5)

Polyphenolic compound	Ethanol extract of *Syzygium cumini*	
	Content (mg/100 g of dry extract)	% RSD
GA	1698.37	2.15
ECA	258.22	1.27
EA	1275.36	2.08
QU	8.97	0.53
TCA	33.02	1.14

*GA* Gallic acid, *ECA* (−)-epicatechin, *EA* ellagic acid, *QU* quercetin, *TCA trans*-cinnamic acid

### Effect on body weight, food and water intake

**Fig. 2:**
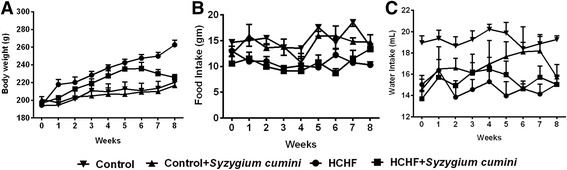
Effect of *Syzygium cumini* seed powder supplementation on body weight (**a**), food (**b**) and water (**c**) intake in high fat diet induced obese rats. Values are presented as mean ± SEM. *n* = 7

The body weight of each rat was noted every day during the experimental period. Significant body weight gain was found in HCHF rats compared to the control rats (Fig. 2, Table 3). *Syzygium cumini seed* powder supplementation showed decreased body weight gain in HCHF diet fed rats. Food and water intake were also decreased in HCHF and HCHF + *Syzygium cumini* rats compared to control and Control + *Syzygium cumini* rats respectively. However, the energy intake of HCHF diet fed rats was higher than the control rats (Table 2).

**Table 3:** Effects of HCHF diet and *Syzygium cumini* seed powder supplementation on body weight, food and water intake and organ wet weight of rats

Parameters	Control	Control + *Syzygium cumini*	HCHF	HCHF+ *Syzygium cumini*
Initial body weight (g)	193.93 ± 1.46a	200.91 ± 3.23a	194.44 ± 1.33a	196.64 ± 1.64a
Final body weight (g)	220.24 ± 6.05a	216.64 ± 4.51a	262.63 ± 5.25b	225.79 ± 3.19a
Food intake /d (g)	15.69 ± 1.48a	14.10 ± 1.32a	13.75 ± 1.12b	10.53 ± 1.21b
Water intake/ d(g)	19.53 ± 0.85a	18.14 ± 1.71a	15.00 ± 1.00b	15.76 ± 0.78b
Energy Intake (kj/day)	282.8 ± 6.3a	256.9 ± 3.8a	382.1 ± 5.1b	300.8 ± 4.9a
Liver wet weight (g/100 g of body weight)	3.09 ± 0.11	3.04 ± 0.09	2.96 ± 0.04	2.70 ± 0.11
Kidney wet weight (g/100 g of body weight)	0.63 ± 0.01	0.57 ± 0.20	0.61 ± 0.01	0.58 ± 0.02
Heart wet weight (g/100 g of body weight)	0.29 ± 0.02	0.27 ± 0.01	0.32 ± 0.02	0.30 ± 0.01
LV of heart (g/100 g of body weight)	0.20 ± 0.01	0.20 ± 0.01	0.22 ± 0.01	0.21 ± 0.01
RV of heart (g/100 g of body weight)	0.03 ± 0.00	0.05 ± 0.01	0.07 ± 0.01	0.05 ± 0.00
Spleen wet weight (g /100 g of body weight)	0.33 ± 0.03	0.36 ± 0.02	0.27 ± 0.01	0.35 ± 0.02
Pancreas (g/100 g of body weight)	0.31 ± 0.11	0.42 ± 0.05	0.38 ± 0.20	0.34 ± 0.02
Fat deposition				
Paritoneal fat (g/100 g of body weight)	0.56 ± 0.07a	0.56 ± 0.10a	2.42 ± 0.17b	1.16 ± 0.13b
Epididymal fat (g/100 g of body weight)	0.44 ± 0.05a	0.71 ± 0.13a	1.18 ± 0.08b	0.87 ± 0.11b
Mesenteric (g /100 g of body weight)	0.34 ± 0.08	0.43 ± 0.04	0.69 ± 0.06	0.69 ± 0.07

Values are presented as mean ± SEM. *n* = 7. One way ANOVA followed by Newman-Keuls post hoc test were done for statistical comparison. Values are considered significance at *p* < 0.05. a vs b, control vs HCHF

### Effect on organ wet weight

Table 3 shows the effect of various treatments on the rats’ organs weight. In comparison to the control group, the liver wet weight was significantly (*p* < 0.05) increased in the HCHF rats. *Syzygium cumini* seed powder (2.5% *w*/w of diet) supplementation significantly (*p* < 0.05) attenuated the wet weight of the liver in the HCHF-treated rats. HCHF-treated rats also showed an increased heart wet weight; *Syzygium cumini* seed powder supplementation normalized the wet weight of the heart. Interestingly the wet weight of pancreas was also increased in HCHF diet fed rats as compared to the control rats. *Syzygium cumini* seed powder supplementation normalized the pancreatic wet weight in HCHF diet fed rats.

### Effect of *Syzygium cumini* seed powder on fat pad deposition and adipose tissue weights

Vivid differences in fat pad deposition levels were observed amongst control, control + *Syzygium cumini*, HCHF and HCHF + *Syzygium cumini* groups. The wet weights of retroperitoneal, mesenteric and epididymal adipose fat pads were markedly increased in HCHF diet fed rats as compared to the control rats. *Syzygium cumini* seed powder supplementation reduced the wet weight fat pad deposition in HCHF diet fed rats significantly, as shown in Table 3.

### Effect of *Syzygium cumini* seed powder on oral glucose tolerance test

**Fig. 3:**
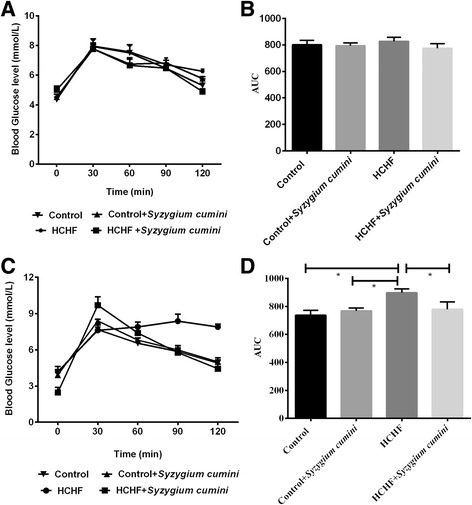
Effect of *Syzygium cumini* seed powder supplementation on oral glucose tolerance test (OGTT) before and after the high fat high carbohydrate diet feeding in rats. **a** OGTT done at the beginning of the study and **b** is the corresponding area under the curve (AUC) values which showed no differences among the groups tested in the study. **c** OGTT done at the end of the study and **d** is the corresponding area under the curve (AUC) values which showed significant differences about glucose utilization among the groups tested in the study. Values are presented as mean ± SEM, *n* = 7. One way ANOVA followed by Newman-Keuls post hoc test were done for statistical comparison. Values are considered significance at *p* < 0.05

The results of oral glucose tolerance test of the control and experimental obese rats are shown in Fig. 3. In normal control rats, maximum elevation in blood glucose level was observed at 60 min after glucose load and declined to near basal level at 120 min, whereas, in HCHF diet induced obese rats, the peak increase in blood glucose level was noticed even after 60 min and remained high over the next 60 min. Interestingly, supplementation of *Syzygium cumini* seed powder to obese rats elicited a significant decrease in blood glucose level at 60 min and beyond when compared with HCHF diet control rats (Fig. 3a).

### Effect of *Syzygium cumini* seed powder on blood glucose, and insulin

Estimation of blood glucose concentration and plasma insulin level in control and experimental obese rats are mentioned in Table 4. There was a significant elevation in blood glucose and plasma insulin concentration (*p <* 0.05) in HCHF diet induced obese rats compared to the control rats. *Syzygium cumini* seed powder supplementation in HCHF diet induced obese rats tended to reduce the elevated blood glucose concentration and normalized the circulating insulin level in blood plasma.

**Table 4:** Effects of high fat diet and *Syzygium cumini* seed powder supplementation on various biochemical parameters in plasma and liver of diet induced obese rats

	Control	Control + *Syzygium cumini*	HCHF	HCHF+ *Syzygium cumini*
Plasma				
AST(U/L)	27.28 ± 4.11a	27.28 ± .46a	55.99 ± 6.58b	30.15 ± 1.93a
ALT(U/L)	30.15 ± 4.85a	25.84 ± 3.15a	50.24 ± 3.46b	44.50 ± 4.11a,c
ALP(U/L)	59.94 ± 3.61a	50.92 ± 8.71a	102.19 ± 7.52b	72.22 ± 6.87a,c
MDA(nmol/mL)	30.48 ± 2.64a	34.02 ± 3.77a	52.38 ± 2.13b	38.79 ± 3.04a
NO(nmol/mL)	8.22 ± 3.71a	9.47 ± 1.07a	17.89 ± 3.25b	10.26 ± 0.87a
APOP (nmol/mL)	272.78 ± 21.71a	294.21 ± 27.52a	546.59 ± 44.17b	336.67 ± 39.28a
Catalase(U/min)	16.33 ± 3.0a	18.67 ± 2.11a	9.50 ± 0.99b	16.50 ± 1.36a
GSH(μg/mg protein)	22.03 ± 2.17a	18.59 ± 0.78a	15.05 ± 0.62b	18.88 ± 0.58a
SOD (U/L)	40.91 ± 3.55a	38.19 ± 4.94a	17.01 ± 3.05b	27.31 ± 3.87a,c
Cholesterol (mg/dL)	166.96 ± 4.70a	155.18 ± 8.31a	313.80 ± 22.52b	233.69 ± 13.70a,c
Triglycerides (mg/dL)	201.48 ± 8.06a	196.75 ± 8.31a	316.89 ± 22.31b	219.93 ± 7.92a
LDL (mg/dL)	35.04 ± 1.83a	39.24 ± 2.70a	42.96 ± 1.66a	45.6 ± 2.72a
HDL (mg/dL)	79.83 ± 9.23a	88.06 ± 6.40 a	229.27 ± 20.74b	145.51 ± 13.72a
Insulin (ng/mL)	0.69 ± 0.08a	1.11 ± 0.40 a	1.85 ± 0.35b	0.82 ± 0.28a
Liver				
MDA(nmol/g tissue)	93.51 ± 4.60a	102.23 ± 5.91a	143.0 ± 9.38b	105.82 ± 8.71a
NO(nmol/ g tissue)	27.43 ± 2.67a	22.66 ± 2.83a	119.96 ± 19.21b	27.19 ± 2.45a
APOP (nmol/g tissue)	726.51 ± 116.48a	701.11 ± 43.15a	1469.37 ± 132.07b	709.84 ± 75.55a
Catalase (U/min/g tissue)	16.33 ± 3.0a	18.67 ± 2.11a	9.50 ± 0.99b	16.0 ± 1.36a
GSH (μg/mg protien)	28.18 ± 3.44a	18.85 ± 1.49a	11.20 ± 0.42b	19.74 ± 3.65a
MPO (U/ g tissue)	0.31 ± 0.05a	0.42 ± 0.04a	1.23 ± 0.12b	0.87 ± 0.16a
SOD (U/g tissue)	38.40 ± 6.74a	44.67 ± 5.53a	15.09 ± 1.80b	28.50 ± 2.35a

Values are presented as mean ± SEM. *n* = 7. One way ANOVA followed by Newman-Keuls post hoc test were done for statistical comparison. Values are considered significance at *p* < 0.05. a vs b, control vs HCHF and b vs c, HCHF vs HCHF+ *Syzygium cumini*

### Serum ALT, AST and ALP activities

In comparison to control rats, HCHF diet fed rats showed elevated plasma ALP, ALT and AST activities along with increased liver wet weights. *Syzygium cumini* seed powder supplementation normalized liver function enzyme activities indicated by the decreased plasma activities of ALP, ALT and AST enzymes (Table 4).

### Plasma and liver antioxidant capacity and lipid peroxidation markers

Table 2 shows the effect of the *Syzygium cumini* seed powder supplementation on plasma and hepatic lipid peroxidation in normal and HCHF diet treated rats. Lipid peroxidation was assessed by malondialdehyde (MDA) formation. Plasma and liver MDA concentrations were significantly (*p* < 0.05) increased in HCHF treated rats as compared to control rats. However, HCHF+ *Syzygium cumini* rats showed decreased plasma MDA concentration compared to HCHF rats. HCHF fed rats also showed increased APOP level in plasma and liver (Table 2). *Syzygium cumini* seed powder supplementation further reduced the rise of APOP level in HCHF + *Syzygium cumini* administered rats (Table 4). Nitric oxide measured as nitrate was also increased in both plasma and liver homogenates of HCHF treated rats compared to control rats. *Syzygium cumini* seed powder supplementation normalized nitric oxide level significantly in both plasma and liver of HCHF administered rats.

### Antioxidant enzymes and glutathione status

The effect of *Syzygium cumini* seed powder supplementation on antioxidant enzyme activities and glutathione redox level in the blood and liver of control and HCHF-treated rats are shown in Table 4. In the plasma, SOD and CAT activities were significantly affected by HCHF treatment. *Syzygium cumini* seed powder supplementation significantly (*p* < 0.05) improved the changes induced in hepatic SOD and CAT activities. In the plasma, HCHF treatment also resulted in significant (*p* < 0.05) depletion of the GSH level when compared to the control rats. *Syzygium cumini* seed powder supplementation in HCHF treated rats restored the GSH levels in plasma compared to the control rats. Hepatic GSH levels were also affected by the HCHF challenge when compared to the control rats. However, mango peel powder supplementation in HCHF treated rats was capable of improving the GSH levels in live as compared to the HCHF treated rats.

### Histological assessment of liver tissue

**Fig. 4:**
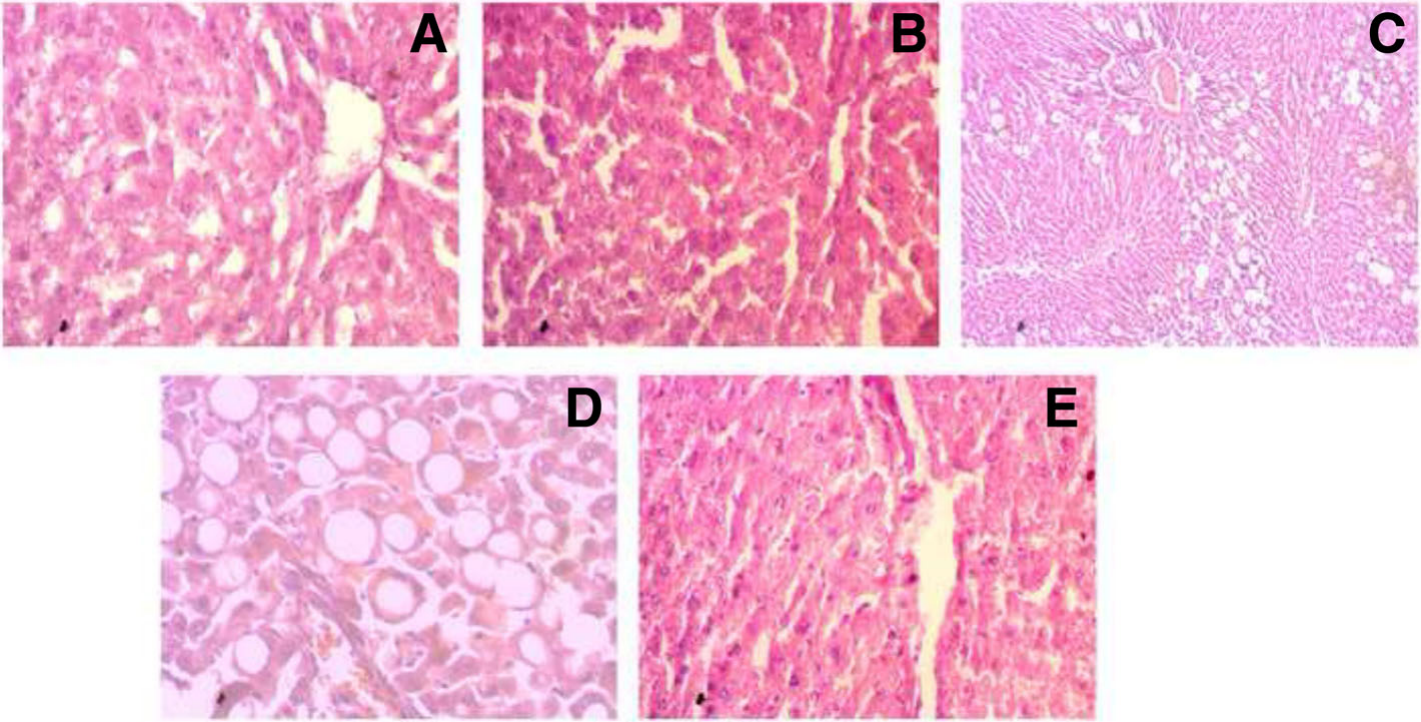
Effect of *Syzygium cumini* seed powder supplementation on hepatic inflammation in high fat diet fed obese rats. **a** Control; **b** Control + *Syzygium cumini* seed; **c** HCHF (low manification); **d** HCHF (high magnification); **e** Fat + *Syzygium cumini* seed. Magnification 40 X

High fat diet fed rats showed increased hepatic lipid deposition and inflammatory cell infiltration compared to control rats (Fig. 4). *Syzygium cumini* seed powder supplementation decreased macrovesicular steatosis and portal inflammation in High fat diet fed rats (Fig. 4). No changes in tissue morphology, inflammatory cell infiltration or macrovesicular steatosis were seen in control + *Syzygium cumini* rats compared to control rats (Fig. 4).

**Fig. 5:**
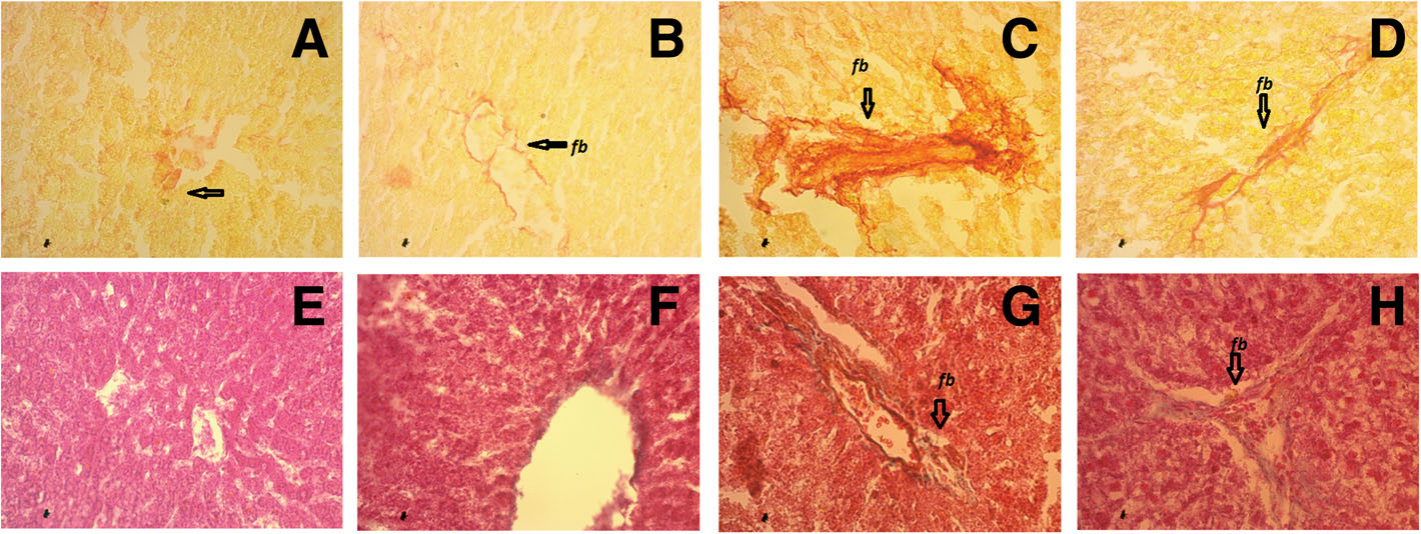
Effect of *Syzygium cumini* seed powder supplementation on hepatic fibrosis in high fat diet fed obese rats. **a**, **e** Control; **b**, **f** Control + *Syzygium cumini* seed; **c**, **g** HCHF; **d**, **h** HCHF+ *Syzygium cumini* seed. Magnification 40 X

**Fig. 6:**
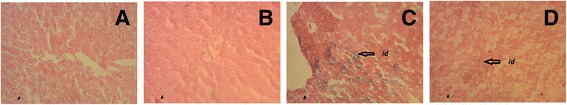
Effect of *Syzygium cumini seed* powder supplementation on hepatic iron deposition in high fat diet fed obese rats. **a** Control; **b** Control + *Syzygium cumini* seed; **c** HCHF; **d** HCHF+ *Syzygium cumini* seed. Magnification 40 X

Sirius red and trichrome milligan staining further confirmed the presence of fibrosis in liver of high fat diet fed rats (Fig. 5). *Syzygium cumini* seed powder supplementation decreased hepatic fibrosis in portal vain area in liver of high fat diet fed rats (Fig. 5). No fibrosis scar was seen in control + *Syzygium cumini* rats compared to control rats (Fig. 5). Moreover, High fat diet fed rats also showed increased hepatic free iron accumulation (Fig. 6). *Syzygium cumini* seed powder supplementation normalized hepatic iron accumulation in liver of high fat diet fed rats. Control rats and control + *Syzygium cumini* rats liver were found free of any iron deposition in this study (Fig. 6).

## Discussion

Obesity is considered as a major health risk and is associated with various health disorders such as insulin resistance, hyperlipidemia, non-alcoholic fatty liver diseases hypertension and cardiovascular dysfunction [28]. Recent shift of dietary behavior from low carbohydrate, high fiber diet to high fat high carbohydrate diet amongst people living in both developed and developing countries is one of the causes of obesity progression [29]. Moreover, sedentary life style also limits the energy expenditure in both young and aged individuals [30]. Obesity treatment is a time consuming and relatively complicated process and there is no easy solution. Moreover, very few drugs are available in the market that are approved by FDA and that should be taken with precaution due to undesirable side effects. The alternative medicine and functional food rich in high polyphenolic component and antioxidants showed promise to reduce body weight gain and related health complications [18, 31]. In this study, we have developed an obese rat model using high fat high carbohydrate diet. These rats mimic human obesity and metabolic syndrome and showed increased body weight gain, fat deposition in peritoneal region, developed glucose intolerance and dyslipidemia. Moreover, high fat diet feeding in rats also increased oxidative stress in liver.

High fat diet feeding is associated with the development of central obesity, insulin resistance, high circulating plasma insulin concentration and non-alcoholic fatty liver [32]. Polyphenolic compound rich food supplement offers a great benefit in obesity related complications [32, 33]. Our investigation found that, high fat diet feeding in rats showed significant glucose intolerance and high level of plasma insulin concentration. Previous report suggests that flavonoid rich extract from *Syzygium cumini* seed has hypoglycemic activity in streptozotocine induced diabetic rats [13]. Our investigation also suggest that Gallic acid and ellagic acid rich *Syzygium cumini* seed powder normalized the impaired glucose tolerance and circulating insulin concentration in high fat diet induced rats. One possible mechanism of *Syzygium cumini* seed powder to be effective on postprandial blood glucose is due to inhibition of  carbohydrate metabolizing-amylase and -glucosidase enzymes or improved insulin resistance in the peripheral tissues such as muscle or adipose tissues [34–36].

Insulin resistance and increased plasma insulin concentration were also observed in obese individual. In this investigation, high fat diet feeding in rats was showed an increased plasma insulin concentration which was further normalized by *Syzygium cumini* seed powder. This effect could be attributed to the capacity of *Syzygium cumini* extract to dual up regulation of both the peroxisome proliferators-activated receptors (PPARα and PPARγ) which were previously reported in liver of streptozotocin induced diabetic rats [13, 35]. Moreover, recent investigations have suggested a role for adipose tissue in the development of insulin resistance. In fact, free fatty acids and various adipokines released from adipose tissue have been involved in the development of insulin resistance [28]. Thus, increased insulin sensitivity to adipose tissues would be another mechanism of improving the insulin resistance in obesity. Adiponectin is one of the adipose-specific adipokine and possesses insulin-sensitizing effects and treatment with adiponectin increases insulin sensitivity in animal models [37, 38]. However, adiponectin concentrations are found low in obese individuals [39]. Thus, body fat mass and insulin resistance are inversely correlated with adiponectin levels. In this study, we have not measured adeponectin level in plasma of high fat diet fed rats. Despite, *Syzygium cumini* seed powder treatment resulted in lowering of fat deposit and improvement of insulin sensitivity in high fat diet fed rats. Previous studies also suggest that restoration of adeponectin level by polyphenol rich extract of *Terminalia paniculata* bark prevented fat deposition in high fat diet-fed rat [40]*.* Further, lowering of peritoneal fat or total fat deposit would be beneficial in obese individual by lowering the adipose tissues derived inflammatory mediators and cytokines production [41].

Oxidative stress is considered as the most crucial events while developing complications in diet induced obesity in rats. Oxidative stress in rats due to high fat diet feeding also increases the glucose intolerance and insulin resistance [42, 43]. Our investigation also revealed that high fat diet increased plasma and tissues level of oxidative stress markers. The results obtained in this study demonstrate that obesity increases lipid peroxidation in hepatic tissues as expressed by increased tissue levels of MDA. High fat diet feeding in rats also increased APOP, nitric oxide level whereas decreased the antioxidant enzyme activities such as SOD and catalase. Milagro et al. showed that obesity is an independent risk factor for increasing lipid peroxidation and decreased activity of cytoprotective enzymes [44]. Moreover, High fat diet feeding in rats decreased the glutathione concentration. Similar findings were also reported in previous studies [42, 45]. *Syzygium cumini* seed powder prevented the rise of plasma oxidative stress markers and restored the antioxidant enzyme activities. Antioxidants such as gallic acid and ellagic acid present in the *Syzygium cumini* seed powder may be responsible for the observed protective mechanism and antioxidant action [18, 46].

Cellular antioxidant enzymes constitute a supportive defense against reactive oxygen species. In the present study, hepatic antioxidant activities of SOD, CAT, GPx and GSH contents were significantly decreased in HCHF diet fed rats as compared to normal diet rats. Another interesting point is that *Syzygium cumini* seed powder normalized the activities of SOD, CAT, GPx and GSH content in hepatic tissue. These results demonstrate that eight weeks HF diet feeding induces oxidative stress to impair the liver tissue. Moreover, hyperlipidemia is considered a major clinical symptom associated with high fat diet feeding in rats. In our study, high fat diet feeding in rats also increased total cholesterol, triglycerides and HDL cholesterol in our study. Chronic dyslipidemia has been characterized as a major risk factor for cardiovascular risk and well as nonalcoholic fatty liver diseases [47]. Previous studies further suggest that high fat diet-induced obesity and abnormal lipid metabolism trigger inflammation, congestion, and nonalcoholic fatty liver disease (NAFLD) leading to hepatic failure marked as boost in AST, ALT, and ALP activity in the serum [48, 49]. Our results showed that consumption of high-fat diet induces fatty liver or hepatic steatosis in rats. *Syzygium cumini* seed powder prevented the rise of plasma total cholesterol and triglyceride levels in our study.

High fat diet feeding in rats further develops inflammation, steatosis and increased fibrosis in liver. Our histological analysis confirmed the inflammatory cells infiltration in liver and lipid accumulation in hepatocytes. Hepatocyte damage and infiltrating inflammatory cells may release inflammatory cytokines and activates hepatic Kuffer cells and hepatic stellate cells (HSCs). Another finding of this study is the increased fibrous tissue accumulation in portal vein and bile duct areas. Activated HSCs are considered the main source of hepatic collagens in fibrosis [50]. Furthermore, an association between IR and mild hepatic iron accumulation has been found particularly in patients with NAFLD. Iron deposits are found in hepatocytes, and/or Kupffer/sinusoidal cells, promoting cell damage. Our investigation also showed increased iron accumulation in liver sections of high fat diet fed rats. *Syzygium cumini* seed powder supplementation ameliorated the inflammatory cells infiltration, fibrosis and iron over load in high fat diet fed rats.

Polyphenol rich extract and pure antioxidants supplementation showed beneficial effect in high fat diet fed obese animals in experimental condition [51, 52]. Some of the phenolic antioxidants such as gallic acid and ellagic acid may prevent pre-adipocyte differentiation to adipocyte [53, 54]. The phenolic antioxidants gallic acid and ellagic acid present in *Syzygium cumini* seed powder could be responsible for the less adipose tissue deposition in high fat diet fed rats. Moreover, gallic acid may also prevent hyperlipidemia and insulin resistance in experimental animals [17, 46]. Previous experiment also suggests that ellagic acid may also regulate fat metabolism in liver and prevent obesity and hyperlipidemia in obese rats [18].

## Conclusion

In conclusion, the present study, for the first time, shows the ameliorative potential of *Syzygium cumini* seed powder on HFD induced glucose intolerance, dyslipidemia, oxidative stress, inflammation and fibrosis in liver. The observed results are attributed to high amount of phenolic antioxidant present in *Syzygium cumini* seed powder. Further studies are warranted to elucidate the mechanism of action mainly focused on fat metabolizing enzyme activities and gene regulation in this model.

## Abbreviations

ALP: Alkaline phosphatase
ALT: Alanine aminotransferase
APOP: Advanced protein oxidation product
AR: Arbutin
AST: Aspartate aminotransferease
BA: Benzoic acid
CA: Caffeic acid
CAT: Catalase
CH: (+)-catechin hydrate
EA: Ellagic acid
ECA: (−)-epicatechin
FA: Trans-ferulic acid
GA: Gallic acid
HCHF: High carbohydrate high fat
HPLC-DAD: High performance liquid chromatography- diode array detection
HQ: Hydroquinone
KF: Kaempferol
MC: Myricetin
MDA: Malondialdehyde
NO: Nitric oxide
PCA: *p*-coumaric acid
QU: Quercetin
RA: Rosmarinic acid
RH: Rutin hydrate
SA: Syringic acid
SOD: Superoxide dismutase
TBA: Thiobarbituric acid
TCA: Trans-cinnamic acid
VA: Vanillic acid
VL: Vanillin
